# Anti-Hypertensive Activity of Novel Peptides Identified from Olive Flounder (*Paralichthys olivaceus*) Surimi

**DOI:** 10.3390/foods9050647

**Published:** 2020-05-18

**Authors:** Jae-Young Oh, Jun-Geon Je, Hyo-Geun Lee, Eun-A Kim, Sang In Kang, Jung-Suck Lee, You-Jin Jeon

**Affiliations:** 1 Department of Marine Life Science, Jeju National University, Jeju 63243, Korea; ojy0724@naver.com (J.-Y.O.); wpwnsrjs@naver.com (J.-G.J.); hond0502@hanmail.net (H.-G.L.); 2 Jeju International Marine Science Center for Research & Education, Korea Institute of Ocean Science and Technology, Jeju 63349, Korea; euna0718@kiost.ac.kr; 3 Department of Seafood and Aquaculture Science, Gyeongsang National University, Tongyeong 53064, Korea; progment@naver.com; 4 Research Center for Industrial Development of Seafood, Gyeongsang National University, Tongyeong 53064, Korea

**Keywords:** antihypertensive, peptide, *Paralichthys olivaceus*, surimi

## Abstract

There is a growing interest in the health benefits of functional foods. A benefit that has been long sought is the control of hypertension through dietary approaches. Hypertension has been implicated as a risk factor for cardiovascular disease and is therefore of clinical significance. Here, we aim to demonstrate the antihypertensive activity of novel peptides derived from surimi, a functional food ingredient made from refined fish myofibrillar proteins. Three peptides, Ile-Val-Asp-Arg (IVDR), Trp-Tyr-Lys (WYK), and Val-Ala-Ser-Val-Ile (VASVI), were isolated from surimi made from the olive flounder (*Paralichthys olivaceus*). Our results show that IVDR, WYK, and VASVI exhibited high Angiotensin I-converting Enzyme (ACE) inhibition activity. These peptides are also shown to increase phosphorylation of protein kinase B (Akt) and endothelial nitric oxide synthase (eNOS), and significantly promote nitric oxide (NO) production in human umbilical vein endothelial cells. Oral administration of the peptides decreased blood pressure in spontaneously hypertensive rats (SHRs), thereby confirming that the peptides derived from surimi perform antihypertensive activity via the Akt/eNOS pathway. These results indicate that surimi made from *P. olivaceus* contains novel antihypertensive peptides that could be used to enhance the health benefits of food ingredients.

## 1. Introduction

Hypertension is a critical risk factor for cardiovascular diseases, especially in populations over the age of 50 years [1]. The global prevalence of hypertension accounts for 9.4 million deaths every year and it is expected that up to 1.58 billion adult patients will suffer from hypertension in 2025 [2].

In recent years, there has been a growing interest in exploring the nutritional value and physiological health effects of functional foods in the diet for prevention and treatment of diseases for a healthy life. Given the clinical significance of hypertension, the anti-hypertensive activity of foods is an important benefit that merits attention and further study [3]. Therefore, many researchers have reported anti-hypertensive effects of beneficial and valuable ingredients in foods [4,5].

Blood pressure and hypertension are immensely influenced by the Angiotensin I-converting enzyme (ACE), which acts through the renin angiotensin aldosterone system [5]. The ACE inhibitory value has been widely used as the primary approach to identifying materials that regulate blood pressure. Previous studies have reported various ACE inhibitory peptides derived from seafood proteins such as sardine, tuna muscle, Alaska pollock skin gelatin, sea horse, and flounder fish [6,7,8,9,10].

Peptides are generally comprised of two to 20 amino acid residues, and their biological function is based on the composition, number, and sequence of amino acids [11]. Production of bioactive peptides occurs during intestinal digestion via the assistance of enzymes. Several activities of bioactive peptides have been reported previously, such as antihypertensive, antioxidant, immunomodulatory, and antimicrobial. While the bioactivities of peptides have been shown to be less than those of synthetic medicine, there have been no reported side effects of natural peptides [12,13].

Surimi is an essential ingredient in processed seafood and has been consumed by the Japanese people since the 1960s. It is made from deboned, minced, washed, and dewatered fish meat to which cryoprotectants such as sorbitol, sucrose, and polyphosphates are added [14]. While the Alaska pollock is a common fish species used to make surimi, other fish species, including the Pacific whiting, threadfin bream, lizard fish, jack mackerel, Atka mackerel, sardine, northern blue whiting, and southern blue whiting, are currently being used for surimi production. Additionally, producers are now beginning to use aquacultured fish species for surimi production [15]. The olive flounder is one such species that is now being used to make surimi in South Korea.

The antihypertensive effect of surimi from olive flounder was subjected to experiment in our previous study, involving human umbilical vein endothelial cells (HUVECs), and systolic blood pressure (SBP) levels in spontaneously hypertensive rats (SHRs). Further, three peptides, IVDR, WYK, and VASVI, were purified and identified from olive flounder surimi [16]. Thus, the present study aims to further investigate the antihypertensive effect of these novel peptides in vitro and in vivo, as well as their therapeutic potential.

## 2. Materials and Methods

### 2.1. Synthesis of Antihypertensive Peptides

The isolated peptides from olive flounder surimi were sequenced, chemically synthesized (Anygen Inc., GwangJu, Korea), and subjected to subsequent experiments (in vitro and in vivo). Amino acid sequencing of the peptides was performed according to the method described by Oh et al. [16]. The amino acid sequences of the synthesized peptides were identified as Isoleucine-Valine-Aspartic acid-Arginine (IVDR; molecular weight: 501.4 Da), Tryptophan-Tyrosine-Lysine (WYK; molecular weight: 495.5 Da), and Valine-Alanine-Serine-Valine-Isoleucine (VASVI; molecular weight: 487.4 Da), respectively.

### 2.2. ACE Inhibitory Activity Assay

The inhibitory activity of ACE was measured using the Dojindo ACE kit-WST (Kumamoto, Japan) in accordance with the manufacturer’s protocols. The principles of the kit have been described in a previous study [17].

The inhibitory ratios were calculated as follows:

Inhibition rate (%) = [(Control − Sample)/(Control − Blank)] × 100(1)

where IC_50_ is the concentration of inhibitor required for 50% inhibition of an enzymatic activity.

### 2.3. Cell Culture and NO Assay

Cultures of human umbilical vein endothelial cells (HUVEC, ATCC* CRL-1730) were incubated at 37 °C in a cell incubator supplied with 5% CO_2_. Cell media compositions were based on those used in a previous study [18]. Furthermore, production of NO was analyzed using Griess reagent (1% sulphanilamide and 0.1% naphthylethylenediamine dihydrochloride in 2.5% phosphoric acid), according to the method described by Ko et al. [18]. HUVEC were seeded in 96-well plates at 1 × 10* cells/mL; after pre-incubation (24 h) the cells were treated without or with three concentrations of peptides (25, 50, and 100 μg/mL) for 3 h. Then the cells were treated with Griess reagent and incubated at 37 °C for 10 min in the dark. The fluorescence was quantified using a microplate reader at 540 nm.

### 2.4. Western Blot Analysis

HUVECs were seeded on 6-well plates at a concentration of 1 × 10* cells/mL. Sixteen hours after seeding, the cells were pretreated with the peptides for 24 h. The experimental procedure was completed as previously described [18]. The primary antibodies (p-Akt, p-eNOS, and β-actin) were used at 1:1000 dilutions and incubated with the membrane at 4 °C overnight. The secondary antibodies were used at 1:3000 dilutions. All antibodies were purchased from Cell Signaling Technology (Beverly, MA, USA).

### 2.5. Animals and Measurement of Blood Pressure

SHRs with tail SBP over 180 mmHg were obtained from SLC Inc. (Shizuoka, Japan). Seven-week-old male, specific pathogen-free SHRs of approximately 300 g body weight were raised under room temperature (25 °C) with a 12-h light/dark cycle. The rats were fed a normal diet and tap water, with ad libitum feeding. All animals were administrated the dissolved samples in saline solution. The rats were divided into five groups comprised of a control group (saline); a group treated with peptide from sardine (positive control); and groups treated with any of three peptides (IVDR, WYK, and VASVI). Each was orally administered to 6 SHRs at a dose of 50 mg/kg which was dissolved in 1 mL saline. SBP was measured by a tail-cuff method before administration and at 3, 6, and 9 h post-administration using a CODA™ blood pressure monitor (Kent Scientific Corp., Torrington, CT, USA). The animal experiment received approval from the Animal Care and Use Committee of the Jeju National University (Approval NO. 2017-0017) and all experiments were performed in accordance with the guidelines.

### 2.6. Statistical Analysis

Data presented in this study are presented as the mean ± standard deviation (SD) of three determinations. The data were analyzed by a one-way ANOVA test (using SPSS ver. 12 statistical software; SPSS Inc., Chicago, IL, USA) by Duncan’s multiple range test. The significant differences were established as ** *p* < 0.05, *** *p* < 0.01, **** *p* < 0.001 (# indicates positive control compared to non-treated group), ** *p* < 0.05, ** *p* < 0.01, *** *p* < 0.001 (* indicates samples group compared to non-treated group or control).

## 3. Results and Discussion

### 3.1. ACE Inhibition Activity of Peptides

Several studies have investigated the relationship between protein intake and blood pressure. The content of amino acids has been found to play an essential role in lowering blood pressure [19]. Our previous study [16] confirmed that surimi manufactured from olive flounder could be used as a beneficial ingredient in dietary protein due to its antihypertensive effect. The study also identified novel peptides derived from surimi. ACE inhibition plays a major physiological role in regulating blood pressure [4]. The current study investigated the ACE inhibition activity of the novel peptides (Table 1). The IC_50_ values for IVDR, WYK, and VASVI were 46.90, 32.97, and 32.66 μM, respectively, thereby indicating remarkable ACE inhibitory activity of each peptide. The majority of ACE inhibitory peptides are of low molecular weight, containing two to 12 amino acids, and are more easily absorbed in the intestinal tract than larger peptides [3,20]. The amino acid sequence of peptides also plays an important role in determining their ACE inhibitory activity [21]. The sequence of ACE inhibitory peptides consists of hydrophobic (Gly, Ala, Val, Leu, Ile, Pro, Phe, Met, and Trp) and aliphatic (Ala, Gly, Ile, Leu, and Val) amino acids at their N-termini [3,22]. The three peptides, IVDR, WYK, and VASVI, were shown to have high ACE inhibitory activity, similar to peptides isolated from food proteins, such as Ala-Ser-Leu (IC_50_ = 102.15 μM) from silkworm pupa, Arg-Val-Cys-Leu-Pro (IC_50_ = 175 μM) from lizard fish, Phe-Gly-Als-Ser-Thr-Arg-Gly-Ala (IC_50_ = 14.7 μM) from Alaska pollock frame, Ala-Leu-Gly-Pro-Gln-Phe-Tyr (IC_50_ = 12 μM) from stone fish, and Ala–Asn–Ser–Glu–Val–Ala–Gln–Trp–Arg and Glu–Ala–Leu–Val–Ser–Gln–Leu–Thr–Arg (IC_50_ = 89.58 and 91.48 μM, respectively) from *Trichiurus lepturus* myosin [3,4,23].

The results of this study reveal that the three peptides are small and are comprised of Ile, Trp, and Val at their N-terminus positions, which are attributes that may contribute to their ACE inhibitory activity.

### 3.2. NO Production in HUVECs Treated with Peptides

**Figure 1:**
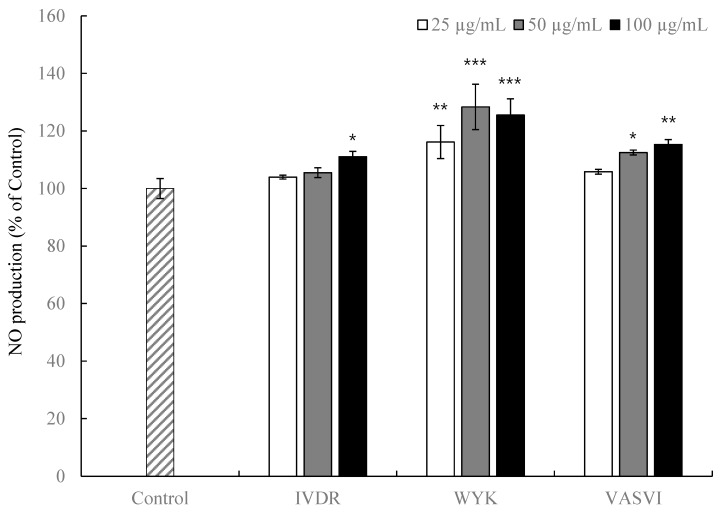
Induction of NO production by the peptides. The HUVECs were treated with various concentrations (µg/mL) of the peptides for 3 h. The values are means ± SD of triplicate experiments. * *p* < 0.05, ** *p* < 0.01, *** *p* < 0.001 as compared to control.

NO, an important factor for regulating blood pressure, has been shown to affect vascular smooth muscle cells underlying the endothelium, and to promote vascular relaxation. Therefore, we measured the NO production in HUVECs using a Griess reagent, intracellular NO indicator [25]. As shown in Figure 1, HUVECs treated with the peptides at 25–100 μg/mL produced more NO compared with the control and did not show toxicity. IVDR treatment increased the production of NO only slightly at the higher concentration of 100 μg/mL. Most notably, the NO production was significantly higher following treatment with WYK and VASVI at concentrations of 25–100 μg/mL. It was reported that antihypertensive peptides derived from food proteins such as milk, eggs, flaxseed, and almond showed induction of NO in HUVECs, indicating a protective role in vascular endothelial function [26,27,28]. Therefore, the three peptides tested here are likely to be potent mediators of endothelium-dependent vasodilation, thus lowering blood pressure.

### 3.3. Antihypertensive Activity of Peptides via the Akt/eNOS Pathway

Endothelial nitric oxide synthase (eNOS), one of the three NOS isoforms (the other two isoforms are inducible NOS (iNOS), and neuronal NOS (nNOS)), is the key enzyme responsible for NO production in blood vessels. eNOS activity increases NO synthesis in HUVECs [29,30] and is activated by direct phosphorylation of Akt [31]. The Akt/eNOS pathway regulates the production of NO. It is therefore anticipated that regulation of the Akt/eNOS pathway will prove effective in the treatment of hypertension [32].

**Figure 2:**
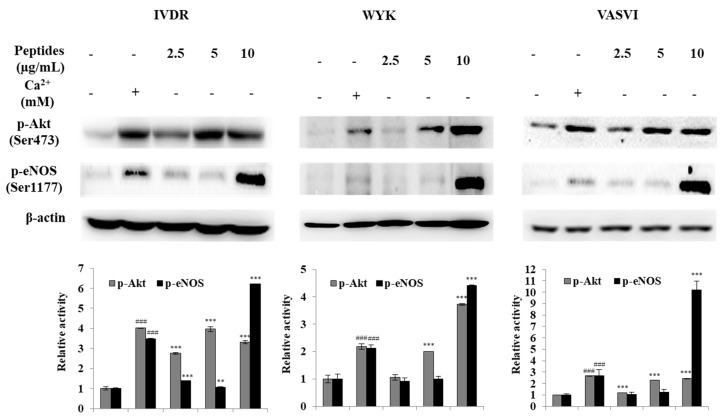
Akt/eNOS phosphorylation level was determined via Western blot analysis in endothelial cells treated with the peptides. The values are means ± SD of triplicate experiments. ^###^ *p* < 0.001 (positive control compared to non-treated group), ** *p* < 0.01, *** *p* < 0.001 (sample group compared to non-treated group).

We observed phosphorylation of Akt and eNOS in HUVECs treated with the peptides, using a Western blot assay. Phosphorylation of Akt and eNOS was profoundly increased in calcium (Ca ****)-treated cells; thus, Ca ** was used as a positive control (Figure 2). Calcium stimulates eNOS activity and plays a role in the activation of the Akt/eNOS pathway [33]. Our results showed that IVDR, WYK, and VASVI treatment at 25, 50, and 100 μg/mL significantly increased the phosphorylation levels of Akt. Furthermore, the phosphorylation of eNOS was particularly induced by the three peptides at the higher concentration of 100 μg/mL. Treatment with each of the three peptides increased NO production through the Akt/eNOS phosphorylation-dependent signaling pathway in HUVECs. Treatment with various antihypertensive drugs has been shown to stimulate phosphorylation of the Akt/eNOS pathway [34,35]. These results suggest that the antihypertensive activity of the peptides is mediated via the Akt/eNOS pathway, which supports the potential use of the peptides as ingredients in nutraceuticals and/or pharmaceuticals.

### 3.4. Antihypertensive Effect of Peptides on SHRs

**Figure 3:**
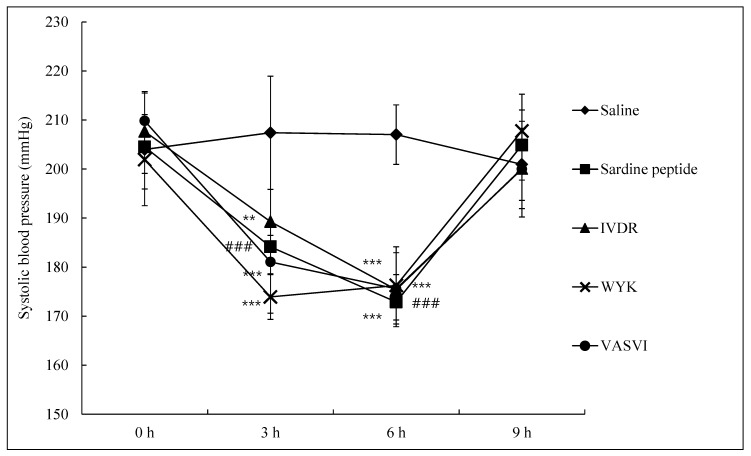
Change in systolic blood pressure (SBP) after oral administration of the peptides in SHRs. (♦) Control (saline); (■) positive control (peptide derived from *Sardino melanostictus*); (▲) IVDR; (×) WYK; (●) VASVI. All the samples including the positive control were orally administrated to the SHRs at 50 mg/kg. The values are means ± SD of triplicate experiments. ^###^ *p* < 0.001 (positive control compared to non-treated group), ** *p* < 0.01, *** *p* < 0.001 (sample group compared to non-treated group).

Changes in systolic blood pressure (SBP) were investigated as a measure of the antihypertensive effect of the three peptides at 3, 6, and 9 h after their oral administration to SHRs. All the samples, including the positive control, were orally administered to the SHRs at 50 mg/kg, and the control group was only saline. In our acute oral administration experiments (Figure 3), the saline group showed slight changes in SBP in the range of 200–210 mmHg. However, at 6 h after the oral administration of the three peptides, SBP was significantly (*p* < 0.001) reduced compared with that in the saline group. SBP also declined considerably after the oral administration of sardine peptide. At 3 h after administration, all the peptides showed rapid decreases of SBP. In particular, treatment with WYK and VASVI elicited low SBP at 173 ± 4 and 181 ± 2 mmHg, respectively, when compared with that in the sardine peptide group at 184 ± 2 mmHg. All the samples except WYK recorded the lowest SBP at 6 h but rose again thereafter. Many studies have revealed that an acute oral administration of antihypertensive compounds showing a rise in SBP after 6 h in SHRs [18,36,37]. Treatment with other antihypertensive proteins and peptides isolated from fish products such as yellowfin sole, cuttlefish, chum salmon, and sardine has also shown reductions of SBP in SHRs [38,39]. Our previous study suggested that surimi consumption was effective at decreasing SBP. The results from our current study now confirm that all three peptides derived from surimi made from olive flounder exert antihypertensive effects in SHRs by reducing SBP [16].

## 4. Conclusions

In this study, the activity and mechanisms of novel anti-hypertensive peptides derived from olive flounder surimi were investigated. These anti-hypertensive peptides had a better ACE inhibitory effect than silkworm pupa, lizard fish, and *Trichiurus lepturus* myosin derived from food protein. Furthermore, NO synthesis, an important factor for regulating blood pressure, is regulated through the Akt/eNOS pathway by the three peptides tested (IVDR, WYK, and VASVI). Those peptides were effective at decreasing SBP in SHRs. Based on the results, we concluded that the novel peptides derived from surimi made from olive flounder, an ingredient in processed seafood, are potentially valuable as antihypertensive ingredients in the nutraceutical or pharmaceutical industries.

## Figures and Tables

**Table 1:** IC_50_ values of ACE inhibitory peptides derived from *Paralichthys olivaceus* surimi.

Peptide	IC_50_ Value (µM)
IVDR	46.90 ± 0.32
WYK	32.97 ± 0.68
VASVI	32.66 ± 1.32
* Sardine peptide (Val-Tyr)	26

IC_50_ values were determined by triplicate experiments. * The IC_50_ value of sardine peptide determined by Matsufuji et al. [24].
